# An Unusual Case of a Gastric Xanthoma: A Case Report

**DOI:** 10.7759/cureus.25026

**Published:** 2022-05-15

**Authors:** Megan R Greenberg, Shashin Shah

**Affiliations:** 1 Gastroenterology, University of South Florida Health Morsani College of Medicine/Lehigh Valley Health Network, Allentown, USA

**Keywords:** endoscopy findings, barrett’s esophagus, upper endoscopy, xanthoma, gastric xanthoma

## Abstract

Gastric xanthomas are rare tumor-like lesions, most commonly occurring in the antral region. We set out to describe a patient with a history of Barrett’s esophagus status post two radiofrequency ablations (RFAs) and an endoscopic mucosal resection (EMR) who developed a gastric xanthoma just below the Z-line with recurrent esophageal metaplasia. Histopathological confirmation of xanthomas are needed to rule out malignancy. While gastric xanthomas themselves are benign conditions, regular follow-up is recommended if there is a high index of suspicion of malignancy or alarming symptoms develop.

## Introduction

Gastric xanthomas are rare tumor-like lesions with an incidence ranging from 0.2-0.8%, most commonly occurring in the antral region. While the lesions themselves are generally incidental asymptomatic findings and benign, they frequently occur in mucosa where pathologic changes such as chronic gastritis, *Helicobacter pylori (H. pylori*) gastritis, intestinal metaplasia, atrophic gastritis, or gastric ulcers are observed [1,2]. These associations have led to the hypothesis that gastric xanthomas are a healing response to local trauma or inflammation. While immunosuppression has been suggested as a possible risk factor for gastric xanthomas, there has been no cited evidence in the literature about how it might be correlated [2]. There is emerging evidence that gastric xanthomas could be useful markers for predicting the development and location of early gastric cancer, and they can sometimes also be associated with dyslipidemia [2,3]. 

We set out to describe a patient with a history of Barrett’s esophagus status post two radiofrequency ablations (RFA) and an endoscopic mucosal resection (EMR), who developed a gastric xanthoma just below the Z-line, which marks the gastroesophageal junction, with recurrent esophageal metaplasia.

## Case presentation

A 57-year-old male with a seven-year history of Barrett’s esophagus status post two RFAs five and three years prior and EMR five years prior presented to our institution for a follow-up for Barrett’s esophagus. He also had a history of fatty liver, non-alcoholic fatty pancreatic disease, chronic gastritis, chronic pain on opioid therapy, and chronic inflammatory demyelinating polyradiculoneuropathy on intravenous immune globulin therapy. His family history was positive for pancreatic cancer in his twin brother.

**Figure 1:**
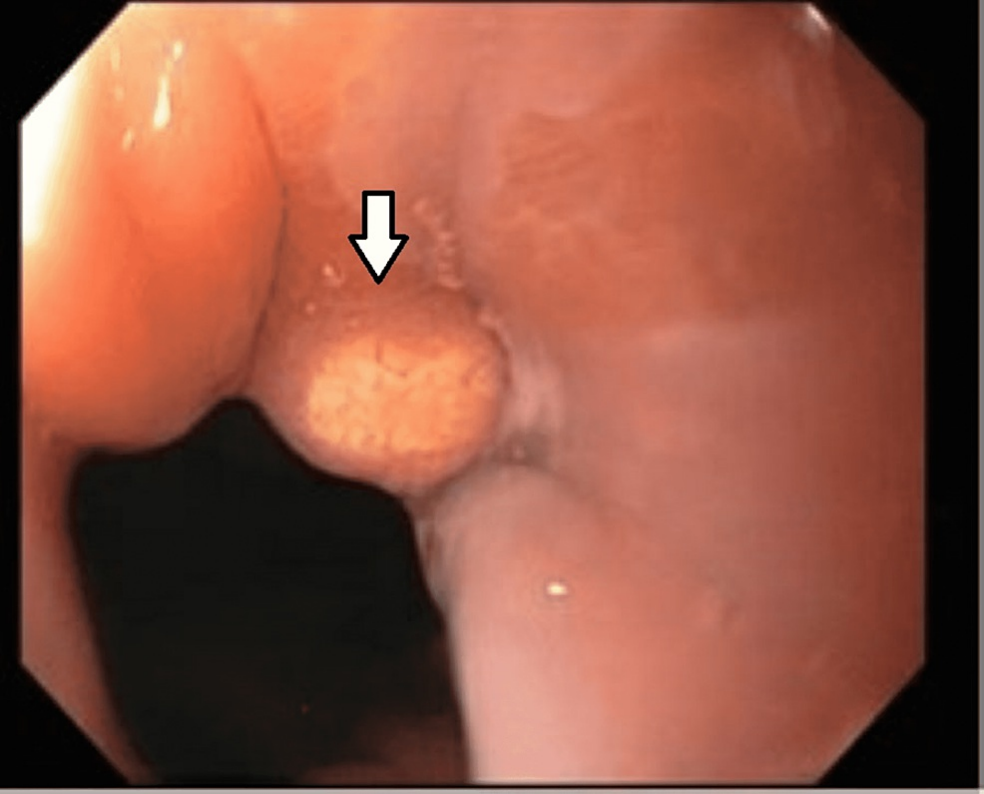
1-2mm yellow-capped nodule biopsy confirmed as a gastric xanthoma

His last esophagogastroduodenoscopy (EGD) one year prior to admission did not show Barrett’s. He reported doing well on his proton pump inhibitor (PPI) dexlansoprazole, his gastroesophageal reflux disease was reportedly controlled, and overall stated this was the “best he had been in a long time”. He had been following a low fermentable oligosaccharides, disaccharides, monosaccharides, and polyols (FODMAP) diet, and had reported recent weight gain. The patient then underwent an EGD, which was notable for a 1-2mm yellow capped nodule below the Z-line (Figure 1).

**Figure 2:**
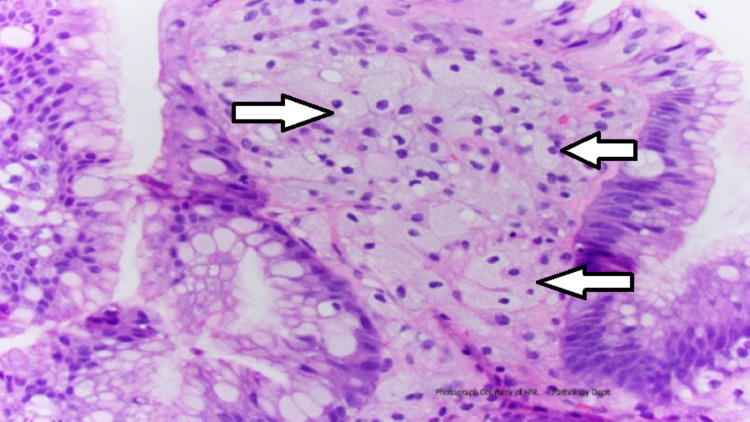
Foamy histiocytic infiltrate in the lamina propria

The Z-line was visualized under narrow-band imaging (NBI), which revealed minimal salmon-colored mucosa suggestive of C0M1 vs. an irregular Z-line. Both abnormalities were biopsied. The EGD also revealed mild non-erosive gastritis localized to the antrum, and the esophagus was otherwise normal in appearance. A biopsy of the nodule revealed columnar/cardiac mucosa with mild chronic inflammation and foamy histiocytic infiltrate in the lamina propria consistent with a gastric xanthoma (Figure 2). 

**Figure 3:**
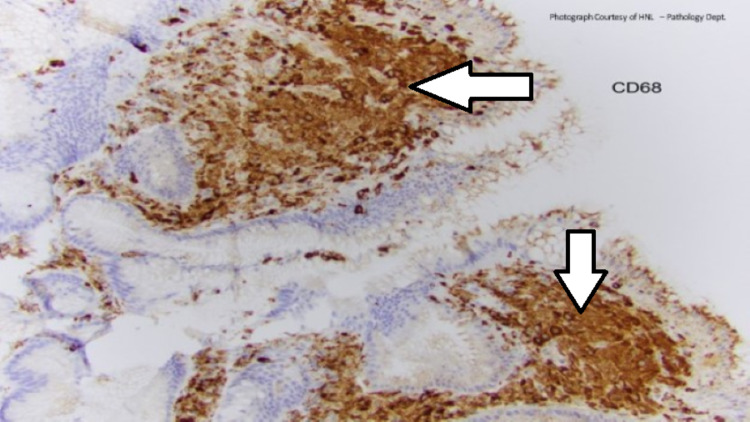
Histiocytic infiltrate in the lamina propria with positive CD68 stain

**Figure 4:**
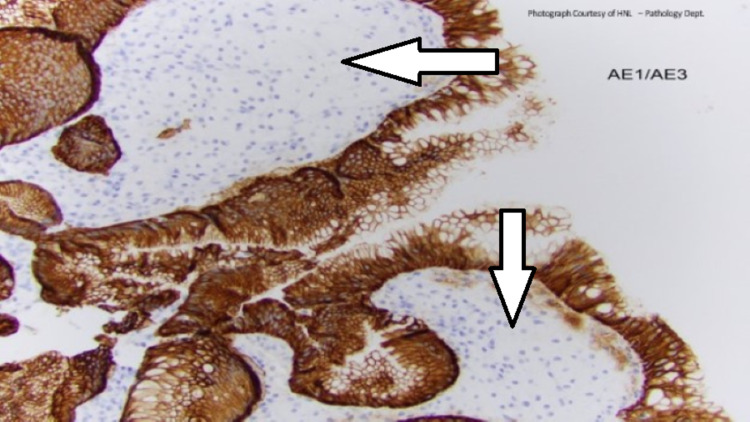
Histiocytic infiltrate in the lamina propria with negative pan-cytokeratin stain

Histiocytic infiltrate in the lamina propria is confirmed by positive CD68 immunohistochemical stain (macrophage marker, Figure 3) and negative pan-cytokeratin stain (epithelial marker, Figure 4). The positive CD68 and foamy macrophages are essential for xanthoma diagnosis, while the negative pan-cytokeratin stain indicates the lack of a gastric tumor. 

The esophageal biopsy revealed squamocolumnar mucosa and gastric cardiac mucosa with chronic inflammation and a single goblet cell consistent with metaplasia and negative for dysplasia. The patient was *H. pylori-*negative. The patient had a follow-up lipid panel done, which was remarkable only for a mild elevation in serum triglycerides to 188, and a mildly low HDL of 34. The patient was advised to continue his PPI and call to schedule his follow-up in six months.

## Discussion

Gastric xanthomas themselves are rare, but the association to date has largely been in the antral region and with gastritis, ulcers, or gastric cancer [1-5]. Xanthomas can occur anywhere in the GI tract [6]. Gastric xanthomas are the most common of the upper gastrointestinal xanthomas, followed by esophageal and duodenal xanthomas [7]. More specifically, gastric xanthomas are most commonly in the antral region but have been seen in the literature in the fundus, cardia, and corpus [1,5,7,8-13]. It is notable that our patient’s gastric xanthoma was near the Z-line, in the setting of a history of Barret’s esophagus and multiple endoscopic procedures, with recurrent metaplasia found on the most recent esophageal biopsy. This association is unique and is worthy of report, as xanthomas have been reported to be a predictive marker for early gastric cancer [5]. To our knowledge, the current case is the only one reported in the literature of a gastric xanthoma specifically noted to be just below the Z-line as well as in association with a history of RFAs. Other reports rarely show gastric xanthomas in the cardia at an unknown distance from the Z-line, as well as esophageal xanthomas near the gastroesophageal junction [7,14].

Histopathological confirmation of xanthomas is needed to rule out malignancy. This is especially true given its association with cancer; it has also been shown that gastric xanthomas are a factor related to the rapid growth of gastric cancer [3]. This case highlights the necessity of searching for accompanying premalignant lesions when a gastric xanthoma is discovered. The location just below the Z-line is important because it supports the hypothesis that gastric xanthomas can be reactive growths in the setting of inflammation or trauma, given the patient’s Barrett’s esophagus and multiple RFAs [2]. The patient’s lipid panel makes dyslipidemia a significant risk factor less likely, and *H. Pylori* was ruled out as a risk factor. While gastric xanthomas themselves are benign, regular follow-up is recommended if there is a high index of suspicion of malignancy or alarming symptoms develop [5]. In our case, the clinician took a conservative approach based on the patient’s history, and the patient was asked to call for a follow-up in six months.

## Conclusions

We described a patient with a history of Barrett’s esophagus and multiple endoscopic procedures, with recurrent metaplasia found on the most recent esophageal biopsy, who was found to have a gastric xanthoma just below the z-line on the most recent endoscopy. It is important to rule out malignancy when these lesions are found due to their risk of being predictive markers of cancer. Frequency of follow-up should be determined by the clinician based on the patient’s individual risk for development of malignancy.
